# Short-term cardiotoxicity and early echocardiographic changes following autologous hematopoietic stem cell transplantation: a single-center retrospective analysis

**DOI:** 10.3389/fcvm.2026.1809002

**Published:** 2026-05-28

**Authors:** Jean El Cheikh, Maria El Tannir, Ali Awada, Ali Tarhini, Nour Moukalled, Iman Abou Dalle, Omar Fakhreddine, Walid Gharzuddin, Ali Bazarbachi, Hadi Skouri

**Affiliations:** 1 Bone Marrow Transplantation Program, Department of Internal Medicine, American University of Beirut Medical Center, Beirut, Lebanon; 2 Research Fellow in the Department of Internal Medicine, American University of Beirut Medical Center, Beirut, Lebanon; 3 Faculty of Internal Medicine, American University of Beirut Medical Center, Beirut, Lebanon; 4 Cardiology Division, Department of Internal Medicine, American University of Beirut Medical Center, Beirut, Lebanon; 5 Cardiology Program, Department of Internal Medicine, American University of Beirut Medical Center, Beirut, Lebanon

**Keywords:** autologous, cardiotoxicity, echocardiography, ejection fraction, hematopoietic stem cell transplant, valvular disease

## Abstract

**Background:**

Autologous stem cell transplantation (ASCT) is a potentially curative treatment for several hematologic malignancies. However, the short-term cardiotoxic effects of conditioning regimens remain underexplored. This study evaluates echocardiographic changes occurring within the first 100 days after ASCT.

**Methods:**

We conducted a retrospective single-center study of 205 lymphoma and Multiple Myeloma patients who underwent ASCT at the American University of Beirut Medical Center (AUBMC) between 2013 and 2022. Echocardiographic parameters before ASCT were compared to those obtained 100 days post-transplant. Conditioning regimens for the lymphoma patients included BEAM (carmustine, etoposide, cytarabine, and melphalan), however for the Multiple Myeloma patients, high-dose melphalan (200 mg/m^2^), and other chemotherapy-based regimens. Primary outcomes were changes in left ventricular ejection fraction (LVEF) and the incident of new valvular disease. Statistical analyses included paired t-tests, McNemar's test, and chi-square tests.

**Results:**

Among 205 patients, 97 (47.3%) had partial remission at transplantation, and 176 (85.9%) had no prior left ventricular (LV) dysfunction. A total of 108 (52.7%) received myeloablative BEAM conditioning. Pre-ASCT, 193 (94.1%) patients had LVEF >50%, compared with 187 (91.2%) at 100 days post-ASCT (*p* = 0.286). The mean LVEF showed a modest but statistically significant decline (*p* = 0.016). The prevalence of valvular abnormalities increased from 86 (42%) to 104 (50.7%) post-ASCT (*p* = 0.073). Pre-existing LV dysfunction was significantly associated with both post-transplant LV dysfunction and valvular disease (*p* = 0.018 and *p* < 0.05, respectively). Male gender was associated with a higher incidence of valvular disease (*p* = 0.024). Conditioning intensity, malignancy type, and prior valvular disease were not significantly correlated with cardiac outcomes. Interestingly, 12 patients with baseline LVEF <50% experienced no cardiac events or ICU admissions post-ASCT; 9 of these demonstrated improved LVEF at follow-up.

**Conclusions:**

ASCT is associated with mild but statistically significant early declines in LVEF and increased valvular abnormalities within 100 days post-transplant. Early post-ASCT echocardiographic surveillance may enable the detection of subclinical cardiotoxicity. Prospective longitudinal studies are warranted to define the long-term cardiac impact of ASCT and its conditioning regimens.

## Introduction

Over the years, extensive research has driven major advances in medical interventions aimed at treating various hematologic malignancies. Among these, the high-dose chemotherapy followed by the autologous stem cell transplantation (ASCT) remains one of the most successful therapeutic strategies for diseases such as multiple myeloma (MM) and certain lymphoma subtypes. Reported overall survival (OS) rates following ASCT range between 89% and 96%, and the procedure has proven highly effective in achieving remission and extending long-term survival (1, 2). Despite its curative potential, ASCT is associated with significant complications, including infections, secondary malignancies, and organ toxicities that can threaten patient outcomes (3). One often underrecognized adverse effect is cardiotoxicity (4, 5). This complication can manifest as cardiomyopathy, heart failure, arrhythmias, right ventricular hypertrophy, and valvular or pericardial disease (6–8).

Echocardiography serves as a cornerstone in the evaluation of cardiac function pre- and post-ASCT, providing insights into chamber dimensions, valvular performance, diastolic filling, and left ventricular ejection fraction (LVEF). The latter being a key parameter for assessing systolic function (6, 8). Moreover, global longitudinal strain (GLS) has emerged as an early marker of subclinical ventricular dysfunction.

Although several studies have explored the prevalence of cardiac events after hematopoietic stem cell transplant, limited data exist on echocardiographic changes and their temporal evolution, particularly following autologous procedures. Monitoring echocardiographic parameters in ASCT recipients is essential for early detection of cardiotoxicity, enabling timely intervention and improved clinical outcomes (9). In this study, all patients underwent a comprehensive cardiac evaluation as part of the pre-ASCT work-up, followed by a systematic echocardiographic reassessment at day +100 post-transplant.

The present study aims to evaluate early echocardiographic alterations occurring within the first 100 days after ASCT and to identify clinical factors associated with these findings. A better understanding of these early changes may contribute to refining post-transplant cardiac surveillance protocols, guiding preventive strategies, and ultimately improving long-term cardiac outcomes in ASCT recipients.

## Methodology

This is a retrospective, monocentric study evaluating and comparing the various alterations in echocardiographic parameters changes pre- and 100 days post-ASCT. The study sample consists of adult patients (age 18 years and above) who underwent their first ASCT at the American University of Beirut Medical Center (AUBMC) between January 2013 and December 2022. Patients were included if they had their echocardiography 30 days prior to and 100 days post-ASCT.

After attaining Institutional Review Board (IRB) approval, Data collection was carried out by reviewing medical records of patients who proceeded to have agreed with a written informed consent for the use of their data in clinical research, as per the local ethics committee and the modified Declaration of Helsinki.

Baseline characteristics including gender, comorbidities, and echocardiographic parameters are presented in Table 1. Valvular disease is defined as pathological involvement of the valves of the heart (Mitral, Tricuspid, Aortic, and Pulmonary), including both stenosis and regurgitations that may denote later for risk of heart failure. In accordance with the American Heart Association (AHA), a reduced LVEF (rLVEF) is defined as a LVEF below 50%. Accordingly, history of LV dysfunction is defined as any previously documented reduction in left ventricular ejection fraction (LVEF <50%) prior to study enrollment. A mild case of Heart Failure with Reduced Ejection Fraction (HFrEF) is categorized as 41%–49%, while moderate to severe HFrEF is seen as below 40%. Moreover, a definition of cardiotoxicity via echocardiography is a 10% decline in LVEF reaching a value below 50%. Additionally, there are cases of possible subclinical cardiotoxicity which is defined as a decline <10% in LVEF to a value <50%, or a relative percentage reduction in GLS by >15%. All readings were analyzed by at least two specialized cardiologists. Pre- and Post- ASCT examinations were performed using the same ultrasound system and the same standardized imaging protocols to minimize inter-study variability.

**Table 1:** Patient and transplant characteristics.

Characteristics of patients	Hodgkin's Lymphoma	Multiple Myeloma	Non-Hodgkin's Lymphoma	Total = 205 (%)	*p* value
Age at transplant (Years)					
<50	36 (41.9)	10 (11.6)	40 (46.5)	86 (41.9)	<0.05
>50	11 (9.2)	75 (63)	33 (27.8)	119 (58.1)	
Gender					
Male	27 (21.6)	59 (47.2)	39 (31.2)	125 (61.0)	0.102
Female	20 (25)	26 (32.5)	34 (42.5)	80 (39.0)	
Disease status at transplant					
Complete Remission (CR)	24 (30)	15 (18.8)	41 (51.2)	80 (39.0)	<0.05
Partial Remission (PR)	15 (15.5)	59 (60.8)	23 (23.7)	97 (47.3)	
in Relapse/Progression	8 (28.6)	11 (39.3)	9 (32.1)	28 (13.7)	
Conditioning Regimen					
BEAM	44 (40.7)	0 (0.0)	64 (59.3)	108 (52.6)	<0.05
Melphalan	2 (2.2)	85 (94.2)	5 (5.4)	92 (44.8)	
Other*	1 (20)	0 (0.0)	4 (80)	5 (2.6)	
History of LV Dysfunction Pre-ASCT					
Yes	4 (33.3)	1 (8.3)	7 (58.3)	12 (5.8)	0.029
No	43 (22.3)	84 (43.5)	66 (34.2)	193 (94.2)	
ASCT Valve Disease					
No	29 (24.4)	48 (40.3)	42 (35.3)	119 (58.0)	0.838
Yes	18 (20.9)	37 (43)	31 (36)	86 (42.0)	
Hypertension					
Yes	5 (10.4)	34 (70.8)	9 (18.8)	48 (23.4)	<0.05
No	42 (26.8)	51 (32.5)	64 (40.8)	157 (76.6)	
Hyperlipidemia					
Yes	2 (6.3)	20 (62.5)	10 (31.3)	32 (15.6)	0.013
No	45 (26)	65 (37.6)	63 (36.4)	173 (84.4)	
Diabetes Mellitus					
Yes	6 (20.7)	18 (62.1)	5 (17.2)	29 (14.15)	0.033
No	41 (23.3)	67 (38.1)	68 (38.6)	176 (85.85)	
Coronoary artery disease					
Yes	1 (11.1)	4 (44.4)	4 (44.4)	9 (95.6)	0.829
No	46 (23.5)	81 (41.3)	69 (35.2)	196 (4.4)	
Smoking					
Yes	19 (32.8)	22 (37.9)	17 (29.3)	58 (28.3)	0.104
No	28 (19)	63 (42.9)	56 (38.1)	147 (71.7)	

*Other include Carboplatin + Etoposide, or Carmustin + Cytarabine + Melphalan, Busulfan + Thymoglobin, or Ifosfomide + Carboplatin + Etoposide.

The malignancies included in the study were conclusive to Hodgkin's Lymphoma, Non-Hodgkin's Lymphoma (NHL), and Multiple Myeloma (MM). Disease status at ASCT was categorized as complete remission (CR), partial remission (PR), and relapsed or progressive (R/R). The conditioning regimens studied were highdose melphalan with a dosage of 200 mg/m^2^. High-dose BEAM consistent of BCNU 300 mg/m^2^, 100 mg/m^2^ Etoposide, 100 mg/m^2^ Ara-C, and 140 mg/m^2^ Melphalan, while some received lower dosage with BCNU 100 mg/m^2^, and 200 mg/m^2^ Thiotepa. The remainder of regimens, which do not fit into either of the two categories above, are shown below and were aggregated together under “Other” due to their low frequency:

- Carboplatin (1,800 mg/m^2)^ and Etoposide (1,200 mg/m^2^)
- Carmustin (300 mg/m^2)^ and Cytarabine (100 mg/m^2^) and Melphalan (140 mg/m^2^)
- Ifosfomide (5 g/m^2^), Carboplatin (550 mg), Etoposide (100 mg/m^2^)

Data analysis was conducted using IBM SPSS version 29. The statistical analysis involved several tests with *p*-values less than 0.05 was considered statistically significant. A paired *T*-test for comparison of continuous variables of echocardiographic parameters before and after ASCT was applied, and a McNemar test to compare the paired categorical variable of LVEF (defined as more or less than 50%) before and after transplant. Additionally, a chi-squared test was prepared to observe patients' baseline characteristics which entail LVEF, new valve disease, cardiac incidences that occurred within 100 days post Day zero of stem cell engraftment with the cardiogenic outcomes of ASCT. Multivariable logistic regression analysis was utilized to identify several independent predictors for occurrence of valvular disease and LVEF decline (<50%). Variable selection for the multivariable models was primarily guided by univariate statistical significance.

## Results

The study cohort comprised 205 patients who met the predefined inclusion criteria and completed a post-ASCT echocardiographic assessment. Of these, 125 (61%) were male. The median age at transplantation was 50.22 ± 14.8 years. Overall, 86 patients (42%) were younger than 50 years, and 119 (58%) were aged 50 years or older. At baseline, 86 (42%) had valvular disease, and 29 (14.1%) had a history of left ventricular (LV) dysfunction prior to transplantation. In addition, 21 patients (10.2%) were receiving angiotensin-converting enzyme inhibitors (ACEIs) or angiotensin II receptor blockers (ARBs) at the time of transplant.

Eighty-five patients (41%) had MM. PR prior to ASCT was documented in 97 patients (47.3%). Regarding conditioning regimens, 102 patients (52.7%) received BEAM, 92 patients (44.9%) received high-dose melphalan, and one patient with pre-transplant LVEF 40%–44% underwent a dose-adjusted BEAM (BCNU 100 mg/m^2^ and thiotepa 200 mg/m^2^). Additional baseline and transplant characteristics are summarized in (Table 1).

### Echocardiographic outcomes

**Figure 1:**
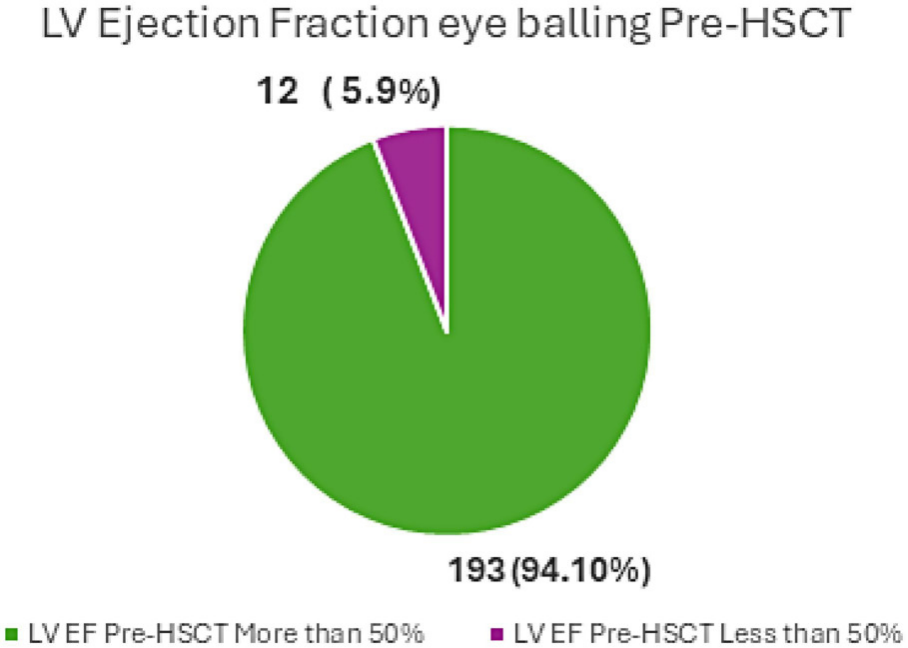
Left ventricular ejection fraction before autologous-HSCT.

**Figure 2:**
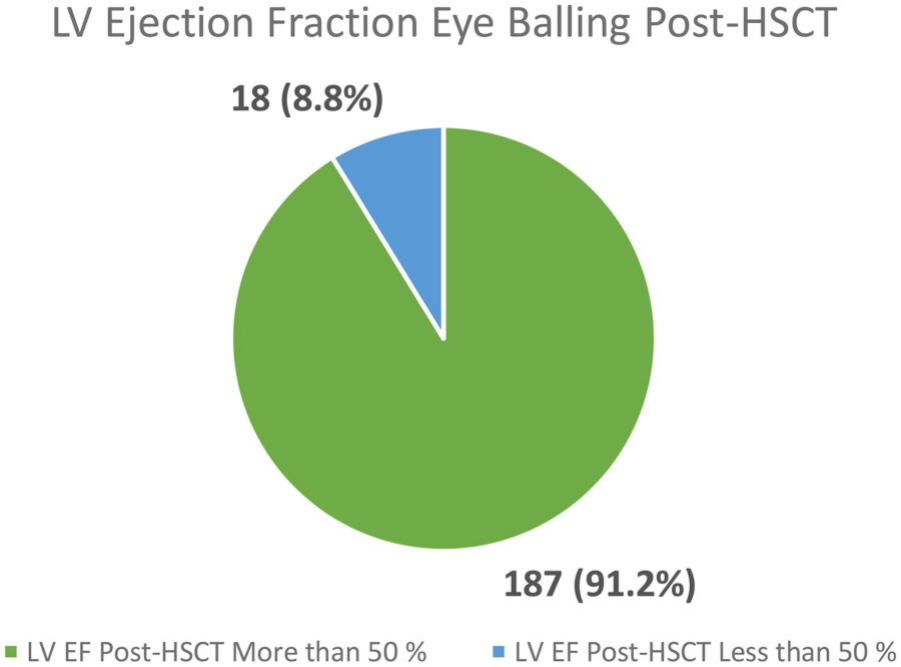
Left ventricular ejection fraction after autologous-HSCT.

Prior to transplantation, 193 patients (94.1%) had LVEF >50%, compared with 187 (91.2%) at day +100 post-ASCT (Figures 1, 2). Although this represents a numerical decline, the McNemar test showed no statistically significant difference (*p* = 0.286) (Table 2).

**Table 2:** Comparison of LVEF and valve disease Pre- and post-ASCT.

LV ejection fraction	Pre-autologous stem cell LV ejection fraction	Post-autologous stem cell LV ejection fraction	McNemar Test
More than 50%	193 (94.1%)	187 (91.2%)	*p* value =0.286
Less than 50%	12 (5.9%)	18 (8.8%)	
LVEF	Pre- Autologous Stem Cell Frequency (percentage)	Post- Autologous Stem cell LV Ejection Fraction Frequency (Percentage)	
65–69%	17 (8.3%)	17 (4.4%)	
60–64%	90 (43.9%)	82 (40.4%)	
55–59%	61 (29.8%)	82 (40.4%)	
50–54%	25 (12.2%)	12 (5.9%)	
45–49%	8 (3.9%)	12 (5.9%)	
40–44%	4 (2%)	3 (1.5%)	
39–35%	0 (0%)	2 (1%)	
30–34%	0 (0%)	1 (0.5%)	
Valve Disease	Pre-ASCT	Post-ASCT	
No	119 (58%)	101 (49.3%)	*p* value = 0.073
Yes	86 (42%)	104 (50.7%)	

The incidence of valvular disease among patients increased from 86 patients (42%) pre-ASCT to 104 patients (50.7%) post-ASCT, demonstrating a clinically notable rise, although the McNemar test indicated borderline significance (*p* = 0.073). The mean LVEF (Simpson method) decreased modestly from 58.32% (SD: 5.77; range 38.2–80) to 57.44% (SD: 5.34; range: 31.5–70) post-ASCT (*p* = 0.016, mean difference 0.924, 95% CI: 0.17–1.67) (Table 3).

**Table 3:** Paired analysis of Pre- and post-ASCT echocardiographic measurements.

Echocardiographic parameter	Pre-ASCT (Mean ± SD)	Post-ASCT (Mean ± SD)	Mean ± SD of difference	*p*-value	95% confidence interval of difference
LV Mass	84.02 ± 22.4	82.12 ± 21.93	1.17 (19.46)	0.442	(−1.83, 4.172)
EDD	46.57 ± 5.3	46.04 ± 5.35	0.48 (6.60)	0.137	(−0.155, 1.12)
ESD	32.43 ± 10.44	31.58 ± 4.59	0.84 (0.737)	0.255	(−0.612, 2.29)
EDV	115.69 ± 31.48	112.8 ± 30.43	2.071 (27.63)	0.187	(−1.32, 6.73)
ESV	49.52 ± 16.18	49.6 ± 15.78	−0.061 (14.27)	0.953	(−2.13, 2.01)
GLS	−19.96 ± 3.12	−19.89 ± 2.74	−0.135 (2.97)	0.55	(−0.58, 0.31)
EF Simpson	58.32 ± 5.77	57.44 ± 5.34	0.924 (5.19)	0.016*	(0.17, 1.67)
S	10.16 ± 8.95	9.65 ± 3.9	0.571 (10.09)	0.447	(−0.908, 2.05)
E	72.19 ± 17.49	69.85 ± 16.04	2.395 (15.14)	0.031*	(0.22, 4.56)
A	72.28 ± 19.12	69.96 ± 17.52	1.92 (16.07)	0.102	(−0.38, 1.23)
E/A	1.04 ± 0.34	1.12 ± 0.76	−0.049 (0.57)	0.242	(−0.13, 0.03)
E′	11.34 ± 3.43	11.13 ± 3.35	0.240 (3.042)	0.286	(−0.20, 0.68)
E/E′	6.36 ± 5.09	6.27 ± 4.78	−0.075 (1.99)	0.618	(−0.37, 0.22)
DT	196.9 ± 48.57	199.98 ± 47.79	−3.399 (57.20)	0.442	(−12.11, 5.31)
TAPSE	19.6 ± 4.87	21.61 ± 3.74	−0.075 (4.68)	0.847	(−0.68, 0.53)
RV S’	14.32 ± 3.02	14.25 ± 2.81	−0.006 (3.081)	0.98	(−0.48, 0.47)
RV E′	11.1 ± 3.1	10.71 ± 2.64	0.462 (3.11)	0.078	(−0.05, 0.97)
SPAP	28.44 ± 5.12	28.04 ± 6.99	0.383 (6.92)	0.669	(−1.40, 2.17)
LA size	26.28 ± 9.84	26.2 ± 8.56	0.005 (0.68)	0.993	(−1.34, 1.35)

* Statistically significant, *p*-value <0.05.

Regarding global longitudinal strain (GLS), the mean pre-ASCT value was −19.96%, compared with −19.89% post-ASCT (mean difference: −0.135%, SD: 2.97, *p* = 0.55, 95% CI: −0.58 to 0.31). For early diastolic trans-mitral flow velocity (*E*), a statistically significant decline was observed from 72.19 cm/s (SD: 17.49) to 69.85 cm/s (SD: 16.04) (*p* = 0.031) (Table 3).

### Predictors of post-transplant cardiac changes

A chi-square analysis (Table 4) revealed that male gender was significantly associated with post-ASCT valvular disease (*p* = 0.024), with 53.8% of male patients developing valvular abnormalities. Additionally, the history of LV dysfunction was strongly associated with post-transplant valvular disease (*p* = 0.018), with 91.3% of these patients developing new or worsening valvular lesions. Among patients aged <50 years and those ≥50 years, the proportions with post-ASCT valvular disease were 41.9% and 57.1%, respectively (*p* = 0.034). For post-transplant LVEF <50%, the proportions were 11.6% and 6.7%, respectively (*p* = 0.317) (Table 4). Among non-diabetic patients, 93 patients (89.4%) developed post-ASCT valve disease, although this association did not reach statistical significance (*p* = 0.099).

**Table 4:** Association between post-ASCT cardiac outcomes (valve disease and LVEF) and patient characteristics.

Patient Characteristics	Total (n)	Post-ASCT valve disease no (%)	Yes (%)	*p*-value	Post-ASCT LVEF > 50% (%)	<50% (%)	*p*-value
Age at transplant				0.034*			0.317
<50 years	86 (42)	50 (58.1)	36 (41.9)		76 (88.4)	10 (11.6)	
≥50 years	119 (58)	51 (42.9)	68 (57.1)		111 (93.3)	8 (6.7)	
Gender				0.024*			0.449
Male	125 (61.0)	69 (68.3)	56 (53.8)		116 (62.0)	9 (50.0)	
Female	80 (39.0)	32 (31.7)	48 (46.2)		71 (38.0)	9 (50.0)	
Hypertension				0.519			0.574
No	157 (76.6)	77 (76.2)	80 (76.9)		142 (75.9)	15 (83.3)	
Yes	78 (23.4)	24 (23.8)	24 (23.1)		45 (24.1)	3 (16.7)	
Hyperlipidemia				0.540			0.318
No	173 (84.4)	85 (84.2)	88 (84.6)		156 (83.4)	17 (94.4)	
Yes	32 (15.6)	16 (15.8)	16 (15.4)		31 (16.6)	1 (5.6)	
Diabetes Mellitus				0.099			0.294
No	176 (85.9)	83 (82.2)	93 (89.4)		162 (86.6)	14 (77.8)	
Yes	29 (14.1)	18 (17.8)	11 (10.6)		25 (13.4)	4 (22.2)	
Coronary Artery Disease				0.235			0.181
No	196 (95.6)	95 (94.1)	101 (97.1)		180 (96.3)	16 (88.9)	
Yes	9 (4.4)	6 (5.9)	3 (2.9)		7 (3.7)	2 (11.1)	
History of LV Dysfunction				0.018*			<0.05*
No	176 (85.9)	81 (80.2)	95 (91.3)		167 (89.3)	9 (50.0)	
Yes	29 (14.1)	20 (19.8)	9 (8.7)		20 (10.7)	9 (50.0)	
Smoking				0.509			1.000
No	147 (71.7)	72 (71.3)	75 (72.1)		134 (71.7)	13 (72.2)	
Yes	58 (28.3)	29 (28.7)	29 (27.9)		53 (28.3)	5 (27.8)	
Cancer Type				0.583			0.179
Hodgkin's	47 (22.9)	26 (25.7)	21 (20.2)		41 (21.9)	6 (33.3)	
Multiple Myeloma	85 (41.5)	39 (38.6)	46 (44.2)		81 (43.3)	4 (22.2)	
NHL	73 (35.6)	36 (35.6)	37 (35.6)		65 (34.8)	8 (44.4)	
Disease status				0.229			0.378
CR	80 (39.0)	37 (36.6)	43 (41.3)		75 (40.1)	5 (27.8)	
PR	97 (47.3)	46 (45.5)	51 (49.0)		88 (47.1)	9 (50.0)	
R/R	28 (13.7)	18 (17.8)	10 (9.6)		24 (12.8)	4 (22.2)	
Conditioning regimen				0.553			0.576
BEAM	108 (52.7)						

* Other include Carboplatin + Etoposide, Carmustin + Cytarabine + Melphalan, Busulfan + Thymoglobin, and Ifosfomide + Carboplatin + Etoposide.

Conversely, the absence of pre-transplant LV dysfunction correlated significantly with a reduction in LVEF post-ASCT (89.3%, *p* < 0.05), as shown in (Table 4). However, conditioning regimen, disease type, and disease status at transplant were not significantly associated with either valvular disease incidence (*p* = 0.553, 0.583, 0.229, respectively) or LVEF <50% (*p* = 0.407, 0.209, 0.421, respectively).

After adjusting for potential confounders, multivariable logistic regression analysis identified several independent predictors for occurrence of valvular disease. These included age (OR: 2.70, 95% CI: 1.44–5.09, *p* = 0.002), gender (OR: 1.91, 95% CI: 1.05–3.47, *p* = 0.033), Diabetes Mellitus (OR: 0.36, 95% CI: 0.14–0.89, *p* = 0.027) and previous history of LV dysfunction (OR: 0.31, 95% CI: 0.12–0.76, *p* = 0.011). No variables remained independently associated with worsening left ventricular ejection fraction (LVEF <50%). These finding are summarized in the Supplementary Table 1.

### Cardiac events and mortality

Among the 12 patients with LVEF <50% before transplantation, 5 had overt LV dysfunction; only one of these received a dose-adjusted conditioning regimen, while the remaining four underwent standard-intensity therapy. All 12 patients tolerated the procedure without cardiac events or mortality during the first year of follow-up. Interestingly, 9 patients of the 12 demonstrated improved LVEF on post-ASCT echocardiography (Table 5).

**Table 5:** Cardiac events and mortality.

Patient	ASCT LVEF Pre/Post	Observation	Valve disease (Pre/Post)	Mortality	Cause of death	Cardiac events in first 100 days post ASCT^2^	CTACE grading
Patient 3	60–64% to 60–64%	No change	—/x	Yes	Pneumonia	No Event	
Patient 9	55–59% to 45–49%	Decrease	—/x	Yes	Sepsis	No Event	
Patient 10	50–54%–60–64%	Improve	x/x	Lost to Follow Up	No Event		
Patient 13	60–64% to 55–59%	Decrease	—/—	Yes	Sepsis	No Event	
Patient 16	55–59% to 60–64%	Improve	—/—	Yes	Sepsis	No Event	
Patient 23	55–59% to 55–59%	No change	—/—	Yes	CMV pneumonitis	No Event	
Patient 41	65–69% to 65–69%	No change	—/—	Yes	Sepsis	No Event	
Patient 79	50–54%–45–49%	Decrease	-/x	Yes	Respiratory failure	No Event	
Patient 85	60–64% to 55–59%	Decrease	x/-	Yes	Cardiac arrest	No Event	5
Patient 133	45–49%	Decrease	—/—	Yes	Sepsis	No Event	
Patient 158	60–64% to 55–59%	Decrease	x/x	Yes	Cardiac arrest	No Event	5
Patient 192	45–49%	Improve	x/x	No	Left chest pain and palpitations	2	

Within the overall cohort, 10 deaths were documented by day +100, predominantly due to sepsis; only two were attributed to cardiac arrest (CTCAE Grade 5). Both patients had LVEF declines from 60%–64% to 55%–59% and no valvular disease on echocardiography. Additionally, one patient with improved LVEF >50% but new-onset valvular disease experienced a Grade 2 cardiac event (chest pain and palpitations). Additional subgroup details regarding baseline comorbidities, conditioning regimens, and outcomes are summarized in (Table 6).

**Table 6:** Characteristics of patient subgroup with LVEF lower than 50%.

Patient	Age	Gender	HTN	Hyperlipidemia	DMT2	CAD	LV Dysfunction	Smoking	Disease status	Conditioning Regimen	LVEF Pre/Post	Valve Disease pre/post	ICU admissions
18	27	—	—	—	—	—	—	—	CR	BEAM	45–49%/55–59%	—/—	—
36	58	—	x	X	X	X	x	—	CR	BEAM	40–44%/30–34%	—/—	
51	58	—	X	—	X	X	x	—	CR	BEAM	40–44%/35–39%	—/—	—
63	57	—	—	X	X	—	—	x	PR	BEAM	45–49%/55–59%	—/—	—
76	69	—	—	—	X	—	X	x	RD	Melphalan	45–49%/45–49%	x/x	—
105	27	—	—	—	—	—	X	—	PR	Modified			
BEAM	40–44%/60–64%	x/x	—										
127	35	—	—	—	—	—	—	—	RD	BEAM	45–49%/55–59%	—/—	—
143	27	—	—	—	—	—	—	—	CR	BEAM	45–49%/55–59%	—/—	—
151	50	—	—	—	—	—	—	x	CR	BEAM	45–49%/55–59%	—/—	—
165	57	x	X	X	X	—	—	—	PR	Melphalan	45–49%/55–59%	—/—	—
186	35	x	—	—	—	—	—	—	CR	BEAM	45–49%/55–59%	x/x	—
192	19	—	—	—	—	—	x	x	CR	BEAM	40–44%/45–49%	x/x	—

## Discussion

This study provides important insights into potential cardiovascular alterations particularly in diastolic parameters, LVEF, and valvular function following ASCT. Although the mean LVEF, as measured by Simpson's method, showed a statistically significant decline following transplantation (*p* = 0.016), the magnitude of this reduction does not meet the established definitions of cancer therapy-related cardiac dysfunction (CTRCD), and is therefore best interpreted as modest rather than clinically significant. These findings are consistent with those of Oliver et al., who reported elevations in cardiac biomarkers and strain abnormalities without significant LVEF reductions (10). Similarly, comparative studies evaluating pre- and post-transplant cardiac function in both autologous and allogeneic stem cell transplant recipients with acute leukemias or multiple myeloma found no significant change in LVEF (11). However, our study is considered as more homogenous because it is performed only in the setting of ASCT.

Nevertheless, a subset of our patients exhibited a decline in LVEF, suggesting that a history of LV dysfunction may be a strong predictor of valvular disease progression following ASCT (*p* = 0.011). This aligns with the findings of Saini et al., who demonstrated that LVEF <50% was an independent predictor of mortality (HR = 2.025, *p* = 0.0835) (12), emphasizing that even mild LVEF reductions can carry prognostic implications in vulnerable populations. However, no sufficient data was collected regarding the specific types of valves involved, as well as the type and severity of valve dysfunction, which we acknowledge as a limitation to our study**.**

Following transplantation, diastolic function also exhibited significant alterations, particularly a decrease in early diastolic transmitral flow velocity (E) (*p* = 0.031), which may reflect subclinical myocardial dysfunction. In Oliver et al.'s study (10), 12.5% of patients experienced a >15% decline in global longitudinal strain (GLS) despite preserved LVEF, supporting GLS as an early marker of myocardial impairment. However, in our cohort, no significant GLS change was observed. Therefore, we cannot conclude that E alone is a reliable indicator for early subclinical dysfunction. Our results can be explained by the differences in follow-up timing, methodological approaches, or patient heterogeneity. Future investigations should further examine the relationship between strain imaging and subclinical cardiotoxicity after ASCT.

Another major observation was the progression of valvular disease, which increased from 42% pre-ASCT to 50.7% post-ASCT. The correlation between baseline LV dysfunction and valvular disease progression (*p* = 0.011) suggests that patients with pre-existing cardiac abnormalities counterintuitively had a lower association of developing a new valvular disease. Although previous studies reported no direct association between valve disease and mortality (13), the increased post-ASCT prevalence warrants close long-term echocardiographic surveillance. Although diabetic patients and those with declined EF appeared to show a protective association against incidence of valvular disease, this finding is likely attributable to differences in clinical management. Patients with diabetes are more likely to receive cardioprotective therapies (ACE inhibitors, beta-blockers, and SGLT2 inhibitors) and to be subjected to more intensive and frequent cardiovascular monitoring throughout the transplant course. These factors may collectively contribute to earlier optimization of cardiac status and reduced likelihood of developing newly detected valvular abnormalities during the follow-up period.

Additionally, the significant association between male gender and valvular complications (*p* = 0.034) underscores the need for further exploration of potential sex-related differences in post-ASCT cardiotoxicity, an area still under-investigated.

Biomarker data reported by Oliver et al. support our echocardiographic findings: at day +100, pro-BNP levels were elevated in 75% of patients, reflecting persistent cardiac strain (10). Although BNP analysis was not performed in this study, the observed structural and functional alterations may correspond to subclinical myocardial stress. Similarly, Vasbinder et al. reported that the combination of fludarabine/melphalan was associated with the highest rate of myocardial complications (7.2% by day 100) (7). In contrast, our analysis did not show a significant correlation between conditioning regimens and cardiac changes, possibly due to differences in methodology, limited follow-up, or smaller subgroups.

Atrial fibrillation (AF) remains a relevant short-term cardiac complication after ASCT, occurring in 2.8%–8.5% of cases, with a five-year cumulative incidence of 6.7% (13). AF incidence was not evaluated in this study, highlighting the need for future research addressing rhythm disturbances in the early post-transplant phase.

Regarding clinical outcomes, ten deaths occurred within the first 100 days, primarily due to sepsis; two cases were attributed to cardiac arrest (CTCAE grade 5). Both patients exhibited mild LVEF declines (from 60%–64% to 55%–59%) without valvular disease. Although causality cannot be inferred given this small sample, these findings emphasize that even minor LVEF reductions may predispose to adverse cardiac events, consistent with the study of Celikci et al. (14) and Saini et al., who identified borderline correlations between arrhythmias and fatal outcomes (HR = 1.88, *p* = 0.0534) (12). Collectively, these results reinforce the necessity of ongoing cardiac monitoring, especially for patients with pre-existing myocardial risk factors.

Despite statistically significant declines in mean LVEF and increased valvular abnormalities, these findings did not correlate with short-term morbidity or mortality. Patients with pre-transplant LVEF <50% tolerated myeloablative conditioning without cardiac events or ICU admissions, and most demonstrated improvement in LVEF by day +100. This apparent dissociation between echocardiographic changes and clinical outcomes may reflect subclinical dysfunction, which precedes overt manifestations by months or years. Factors such as low baseline comorbidity, preserved cardiac reserve, use of cardioprotective medications, such as angiotensin converting enzyme inhibitors (ACEI), beta-blockers, and lipid-lowering statins and short follow-up duration may have mitigated overt cardiac complications. These observations underscore the importance of systematic echocardiographic follow-up to detect early dysfunction predictive of long-term risk (15).

The tolerance of ASCT in patients with impaired LVEF has recently attracted interest. Several reports suggest that ASCT can be performed safely in this subgroup, particularly with dose-reduced or tailored conditioning regimens. Hessling et al. demonstrated the feasibility of reduced-intensity protocols in elderly patients with cardiac comorbidities (16), while Kim et al. showed that reduced-intensity conditioning (RIC) allogeneic transplantation in high-risk patients with less cardiac stress (17). Further emphasizing the cardiac implications of conditioning intensity, El-Cheikh et al. retrospectively analyzed 310 allogeneic-HSCT recipients at AUBMC and identified a small but significant decline in mean left ventricular ejection fraction (59.1%–58.4%, *p* = 0.037), particularly in patients receiving haploidentical transplantation, cyclophosphamide-containing, or sequential conditioning regimens (18). Although largely subclinical, the proportion of patients with LVEF <50% doubled post-transplant (3.1%–6.7%), underscoring the need for continued echocardiographic surveillance in regimens utilizing cyclophosphamide-based RIC or post-transplant cyclophosphamide prophylaxis. Similarly, Oyama et al. reported improved outcomes in systemic sclerosis patients treated with autologous non-myeloablative SCT using cyclophosphamide and ATG without total body irradiation (19). In our cohort, patients with reduced pre-transplant LVEF tolerated conditioning without cardiac complications comparable to findings from Markiewicz et al. (20) and Neto et al. (21).

Few studies have specifically examined echocardiographic alterations between baseline and day +100 post-ASCT, representing a significant gap in literature. This work offers several advantages that strengthen the validity and clinical relevance of its findings. By concentrating on a single-center ASCT population with standardized pre-transplant and day +100 echocardiograms, we were able to perform comparisons under consistent imaging protocols, limiting measurement variability and enhancing internal reliability. Additionally, the cohort is relatively large for cardio-oncology in this setting (*n* = 205) and reflects contemporary practice across a decade, improving the precision and applicability of the estimates. Importantly, the echocardiographic evaluation encompasses systolic function by Simpson LVEF, multiple indices of diastolic function, right-sided measures, strain, and valvular assessment, with detection of subclinical alterations without relying on LVEF alone. A predefined day +100 window ties these imaging changes to an immediately relevant clinical phase after ASCT, allowing us to relate echocardiographic shifts to short-term outcomes. Moreover, inclusion of patients with baseline LVEF <50%, a group commonly excluded from trials, provides pragmatic evidence that many can tolerate ASCT without early cardiac events and may even demonstrate LVEF improvement.

The main limitation of our study is the short follow-up duration, which precludes assessment of the persistence and clinical relevance of early cardiac changes. In addition, the single center setting may limit generalizability and the lack of a control group emphasize that our findings should be interpreted as associative rather than causal. Additionally, Inter-observer data variability during echocardiography assessment was not formally addressed, which we acknowledge as a limitation. Also, the limited data on the history of previous chemotherapy exposure and thoracic irradiation may confound the observed cardiac outcomes. Furthermore, the absence of systematic data on valvular abnormalities represents an important limitation, as it restricts the comprehensiveness of cardiac evaluation and may influence the interpretation of our findings. Finally, selecting variables for the multivariable models was primarily guided by univariate statistical significance. We acknowledge this as a methodological limitation, and a more rigorous approach would be required.

Nevertheless, the study's strength lies in its systematic echocardiographic assessment at predefined time points, providing a robust characterization of early myocardial responses to ASCT.

Future research should aim to establish the long-term prognostic significance of early echocardiographic alterations, integrating strain imaging and cardiac biomarkers into routine post-transplant evaluation to improve early detection of subclinical cardiotoxicity. In addition, longitudinal echocardiographic follow-up and advanced imaging approaches are needed to better elucidate the mechanisms underlying ASCT associated cardiac dysfunction in larger, multi-center cohort studies.

Finally, the assessment of preventive cardioprotective strategies and the implementation of risk-adapted cardiac surveillance protocols may help mitigate cardiovascular morbidity and optimize clinical outcomes in patients undergoing ASCT.

## Conclusion

In conclusion, this study adds valuable evidence to the limited body of literature addressing ASCT associated cardiotoxicity, underscoring the importance of detecting subclinical cardiac alterations and monitoring the progression of valvular disease in the early post-transplant period. Our findings highlight that even mild echocardiographic changes may precede overt cardiac dysfunction, emphasizing the need for systematic surveillance in this patient population.

## Data Availability

The original contributions presented in the study are included in the article/Supplementary Material, further inquiries can be directed to the corresponding author.

## Glossary

LV mass (left ventricular mass): A measurement of the total mass of the left ventricle of the heart. an indicator of left ventricular hypertrophy (LVH).
LVEF (left ventricular ejection fraction): The percentage of blood ejected from the left ventricle with each contraction. A normal LVEF is generally considered to be between 55% and 70%. Reduced LVEF can indicate heart failure or other cardiac problems
EDD (end diastolic diameter): The diameter of the left ventricle at the end of diastole.
ESD (end systolic diameter): The diameter of the left ventricle at the end of systole.
EDV (end diastolic volume): The volume of blood in the left ventricle at the end of diastole.
ESV (end systolic volume): The blood remaining in the left ventricle at the end of systole.
GLS (global longitudinal strain): A measure of the deformation of the left ventricle during contraction, specifically how much the myocardial fibers shorten in the longitudinal direction. GLS is a sensitive marker of myocardial function and can detect subtle abnormalities even when the ejection fraction is normal. More negative values typically indicate better function.
EF Simpson ejection fraction (Simpson method): It's considered more accurate than visually estimating EF, especially in cases of regional wall motion abnormalities.
*S* (peak velocity of systolic pulmonary vein flow): The maximum velocity of blood flow in the pulmonary veins during ventricular systole. It reflects left atrial function and can be affected by left ventricular dysfunction.
*E* (peak velocity of early diastolic transmitral flow): The maximum velocity of blood flow through the mitral valve during early diastole.
*A* (peak velocity of late transmitral flow): The maximum velocity of blood flow through the mitral valve during late diastole, caused by atrial contraction.
*E*/*A* (Ratio of early diastolic filling velocity to late diastolic filling velocity): Changes in this ratio can indicate diastolic dysfunction.
*E*′ (early diastolic tissue doppler velocity): The velocity of the mitral annulus moving towards the apex of the heart during early diastole. It reflects the rate of left ventricular relaxation.
*E*/*E*′ (Ratio of *E* to *E*′): The ratio of mitral inflow E velocity to mitral annular *E*′ velocity. It's an estimate of left ventricular filling pressure. Elevated *E*/*E*′ ratios often indicate elevated left ventricular filling pressures and diastolic dysfunction.
DT (deceleration time): The time it takes for the early diastolic transmitral flow (E wave) to decelerate to zero. It's another measure of diastolic function, with shorter DTs often seen in restrictive filling patterns and longer DTs in impaired relaxation.
TAPSE (tricuspid annular plane systolic excursion): A measure of right ventricular systolic function. It measures the amount the tricuspid annulus moves towards the apex of the heart during systole. A lower TAPSE indicates impaired right ventricular function.
RV *S*′ (right ventricular systolic velocity): The peak systolic velocity of the tricuspid annulus, measured using Tissue Doppler Imaging. It's another measure of right ventricular systolic function.
RV *E*′ (right ventricular early diastolic velocity): The early diastolic velocity of the tricuspid annulus, measured using Tissue Doppler Imaging. It reflects right ventricular relaxation.
SPAP (systolic pulmonary artery pressure): An estimate of the pressure in the pulmonary artery during systole, derived from the tricuspid regurgitation jet velocity. Elevated SPAP indicates pulmonary hypertension.
LA size (left atrial size): Measurement of the left atrium, usually in terms of volume or diameter. Enlarged left atrial size can be indicative of chronic elevation of left atrial pressure, often due to left ventricular diastolic dysfunction or mitral valve disease.
