# Supplementary material for: Short-term cardiotoxicity and early echocardiographic changes following autologous hematopoietic stem cell transplantation: a single-center retrospective analysis
Source: Front Cardiovasc Med. 2026 May 28;13:1809002. doi: 10.3389/fcvm.2026.1809002 (PMC13253228; doi:10.3389/fcvm.2026.1809002)
Supplement: Supplementary file 1 [file Datasheet1.docx]

# Supplementary tables:

Table 1: Multivariable Logistic Regression Analysis of Factors Associated with the Occurrence of valvular diseases after ASCT.

| Variable | Odds Ratio | 95% CI (Lower–Upper) | p-value |
| --- | --- | --- | --- |
| Age | 2.70 | 1.441–5.093 | 0.002* |
| Gender | 1.91 | 1.054–3.472 | 0.033* |
| History of LV Dysfunction | 0.31 | 0.128–0.763 | 0.011* |
| Diabetes Mellitus | 0.36 | 0.148–0.892 | 0.027* |

Table 2: Multivariable Logistic Regression Analysis of Factors Associated with decline in EF<50% after ASCT.

| Variable | Odds Ratio | 95% CI (Lower–Upper) | p-value |
| --- | --- | --- | --- |
| Age | 4.25 | 0.980–18.457 | 0.053 |
| Gender | 0.41 | 0.124–1.403 | 0.158 |
| History of LV Dysfunction | 0.47 | 0.122–1.881 | 0.291 |
| Diabetes Mellitus | 0.285 | 0.049–1.665 | 0.163 |
| Coronary Artery Disease | 0.126 | 0.015-1.078 | 0.059 |
